# AT1R/GSK-3*β*/mTOR Signaling Pathway Involved in Angiotensin II-Induced Neuronal Apoptosis after HIE Both In Vitro and In Vivo

**DOI:** 10.1155/2020/8864323

**Published:** 2020-12-22

**Authors:** Wei Si, Banghui Li, Cameron Lenahan, Shirong Li, Ran Gu, Hao Qu, Lu Wang, Jiapeng Liu, Tian Tian, Qian Wang, XikeWang, Xiao Hu, Gang Zuo

**Affiliations:** ^1^Department of Neurology, Guizhou Provincial People's Hospital, Guiyang 550002, China; ^2^Burrell College of Osteopathic Medicine, Las Cruces, NM 88003, USA; ^3^Department of Paediatrics, Guizhou Provincial People's Hospital, Guiyang 550002, China; ^4^Department of Neurosurgery, Taicang Hospital Affiliated to Soochow University, Taicang, Suzhou, Jiangsu 215400, China

## Abstract

**Objective:**

The focus of the present study is to evaluate the effects of Angiotensin II (Ang II) on neuronal apoptosis after HIE and the potential underlying mechanisms.

**Methods:**

Primary neonatal rat cortical neurons were used to study the oxygen-glucose deprivation (OGD) cell model. The expressions of Ang II, AT1R, GSK-3*β*, p-GSK-3*β*, mTOR, p-mTOR, Bax, Bcl-2, and cleaved caspase-3 were detected via western blot. IF and flow cytometry were used to evaluate neuronal apoptosis. Hypoxic-ischemic encephalopathy (HIE) was established to evaluate the therapeutic effects of Ang II in vivo. Cerebral infarction areas were detected by 2,3,5-Triphenyltetrazolium chloride staining. The righting and geotaxis reflexes were also recorded. In addition, Fluoro-Jade C staining and TUNEL staining were performed to evaluate neuronal degeneration and apoptosis.

**Results:**

Ang II significantly increased the rate of neuronal apoptosis, upregulated the expression of cleaved caspase-3, and downregulated Bcl-2/Bax ratio after OGD insult. For vivo assay, the expressions of endogenous Ang II and AT1R gradually increased and peaked at 24 h after HIE. Ang II increased NeuN-positive AT1R cell expression. In addition, Ang II increased the area of cerebral infarction, promoted neuronal degeneration and apoptosis, aggravated neurological deficits on righting and geotaxis reflexes, and was accompanied by increased expressions of phosphorylated GSK-3*β* and mTOR. The application of valsartan (Ang II inhibitor) or SB216763 (GSK-3*β* inhibitor) reversed these phenomena triggered by Ang II following HIE.

**Conclusion:**

Ang II increased neuronal apoptosis through the AT1R/GSK-3*β*/mTOR signaling pathway after experimental HIE both in vitro and in vivo, and Ang II may serve as a novel therapeutic target to ameliorate brain injury after HIE.

## 1. Introduction

Hypoxic-ischemic encephalopathy (HIE) represents a subcategory of ischemic stroke and is a devastating brain disease in neonates, which could lead to disability or even death. The incidence of HIE was as high as 1,500 per million live births [1]. Presently, several studies found that HIE pathogenesis included oxidative stress, mitochondrial injury, apoptosis, and autophagy [2]. Among them, neuronal apoptosis in HIE has attracted substantial attention and has been extensively studied. Wu et al. reported that PKM2 was involved in HIE-induced neuronal apoptosis [3], and that chloroquine could impair neuronal apoptosis in hypoxic neurons [4]. However, the mechanism of apoptosis in HIE has yet to be fully elucidated.

Angiotensin II (Ang II), a stress-related neuropeptide, participated in the regulation of water and sodium homeostasis [5]. As a main component of the renin-angiotensin system (RAS), it was found that Ang II was expressed in the brain and was involved in the pathogenesis of neurological diseases [6–8]. Of note, it was indicated that the RAS in the brain was activated postneuronal injury, and the blockade of RAS exerted neuroprotective effects [9]. It was widely accepted that Ang II signaling was transmitted by binding to its receptors, namely, Ang II type 1 receptor (AT1R) and AT2R. The AT1R and AT2R exert opposite functions after binding Ang II. For example, inhibition of AT1R could prevent retinal neural damage and elevate AT2R function [10]. In a study of reperfusion myocardial infarction, inhibition of AngII/AT1R signaling led to the upregulation of AT2R, which reduced infarction area, but had no effect on neuronal apoptosis. In this study, apoptosis was mediated by AT1R instead of AT2R [11]. Moreover, Moudgil et al. suggested that increased AT2R expression could promote cardioprotection without affecting apoptosis [12]. More importantly, because of the relatively low cellular expression, the functions of AT2R are more difficult to study compared to AT1R [13]. Therefore, we inferred that AT1R, rather than AT2R, has a dominant role in neuronal diseases. AT1R antagonists can inhibit neuroinflammation and dopaminergic neuronal death and can reduce the toxicity of 6-hydroxydopamine to neurons in Parkinson's disease [14, 15]. Shindo et al. reported that blockade of AT1R in Alzheimer's disease can increase spatial memory [16] and promote hippocampal neurogenesis [17], but inhibition of AT1R could attenuate ischemic brain injury [18, 19]. Combined with previous studies, we speculated that Ang II/AT1R might be involved in neuronal injury, which deserves further investigation.

Glycogen Synthase Kinase-3*β* (GSK-3*β*) regulates a variety of important biological processes, including glucose homeostasis [20], energy metabolism [21], and apoptosis [22], and its dysregulation was associated with neurological diseases, such as Alzheimer's disease [23] and Parkinson disease [24]. Previous studies have shown that the mammalian target of rapamycin (mTOR), a modulator for glucose metabolism, is an important regulator of GSK-3*β*, a mediator of glycolysis [25], cell apoptosis [26], and oxidative stress [27]. In addition, GSK-3*β*/mTOR signaling has been shown to regulate autophagy [28], but inhibition of GSK-3*β*/mTOR could induce synaptogenesis and axonal repair in a neonatal rat model of HIE [29]. It was suggested that GSK-3*β* was a downstream target for Ang II [30]. However, it remains unclear whether Ang II regulates GSK-3*β*/mTOR activity.

**Figure 1:**
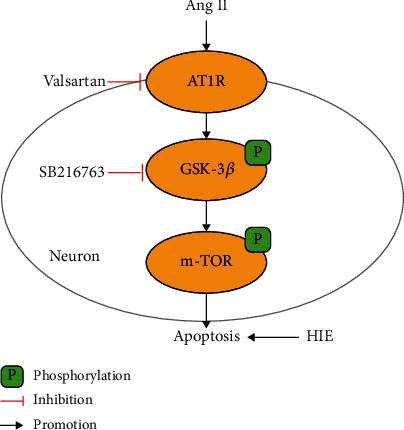
Graphic illustration for Ang II mediated neuronal apoptosis in Hypoxic-ischemic encephalopathy.

In this present study, we explored the role of Ang II in HIE. We demonstrated, for the first time, that the AT1R/GSK-3*β*/mTOR signaling pathway was involved in AngII-induced neuronal apoptosis after HIE (Figure 1).

## 2. Methods and Materials

### 2.1. Cell Culture

Primary neuronal cells were used for in vitro analysis, and the isolation and culture procedures were performed according to our previous study [31]. The isolated cells were identified via morphology examination and MAP2 staining.

### 2.2. Immunofluorescence Staining (IF)

For cell experiments, the IF staining was described in our previous reports [32]. In brief, 1 × 105 neuronal cells were plated on glass coverslips for 24 h, fixed using 4% formaldehyde, and penetrated with 0.1% Triton X-100. Next, 5% (*w*/*v*) goat serum was used to block nonspecific signals at room temperature for 1 h, and then anti-MAP2 antibody (1 : 100, Abcam, UK) was incubated overnight at 4°C. For tissue examination, the procedures were similar to that of the cell experiments. Briefly, the brain tissues were cut into 5 *μ*m thick slices, then the slices were fixed, penetrated, and blocked. The primary antibodies against AT1R (1 : 2000, Abcam, UK), NeuN (1 : 200, Abcam, UK), Fluoro-Jade C (FJC, 1 : 100, Abcam, UK), and fluorescein-dUTP (dUTP, 1 : 100, Abcam, UK) were added and maintained overnight at 4°C. Afterward, the coverslips were incubated with the corresponding HRP-conjugated secondary antibodies (1 : 200, Gen Tex, USA), counterstained with DAPI, and observed under a laser confocal microscope (Olympus, Tokyo, Japan).

### 2.3. Oxygen-Glucose Deprivation (OGD) Cell Model

The OGD cell model was established according to a previous report [34]. The primary neuronal cells were cocultured with glucose-free Earle's Balanced Salt solution (Sigma-Aldrich, MO, USA) for 1 h to establish the OGD cell model. To explore the effect of Ang II on neuronal function, the OGD pretreated cells were treated with Ang II (10 *μ*mol/l, Sigma-Aldrich, MO, USA) or vehicle, with normal cells serving as the sham group.

### 2.4. Flow Cytometry

Cell apoptosis was detected according to our previous report [31] using Annexin V/Propidium iodide staining kit (BD Bioscience, CA, USA). In brief, after Ang II treatment, 1 × 105 neuronal cells were washed using cold PBS, stained with Annexin V and Propidium iodide, and analyzed using a flow cytometer (BD Bioscience, USA).

### 2.5. Western Blot

The expression of Ang II, AT1R, GSK-3*β*, p-GSK-3*β*, mTOR, p-mTOR, Bax, Bcl-2, and cleaved caspase-3 at the protein level was determined by western blot as previously described [34]. The primary antibodies against Ang II (1 : 1000, Abcam, UK), AT1R (1 : 2000, Abcam, UK), GSK-3*β* (1 : 500 Abcam, UK), p-GSK-3*β* (1 : 1000, Abcam, UK), mTOR (1 : 1000, Abcam, UK), p-mTOR (1 : 1000, Abcam, UK), Bax (1 : 500, Abcam, UK), Bcl-2 (1 : 1000, Abcam, UK), cleaved caspase-3 (1 : 500, Abcam, UK), and *β*-actin (1 : 3000, Abcam, UK) were incubated overnight at 4°C. The band density was evaluated by Quantity One (Bio-Rad, USA) with *β*-actin as the reference gene.

### 2.6. Animals

Sprague Dawley rat pups and their mothers were obtained from Guizhou Laboratory Animal Engineering Technology Center (China). The rats were maintained in a 12/12 h light and dark cycle, with ad libitum access to water. The 8-day old pups were used for the HIE model. The rat HIE model was performed as previously described [35]. Briefly, the pups were anesthetized using 3% isoflurane (Sigma-Aldrich, USA), and a sagittal incision was made on the neck 4 mm lateral to midline. Then, the right carotid artery was isolated, ligated, and severed. After completing the previous steps, the skin was sutured. The entire procedure was performed on a thermostable blanket and kept at 37°C. Regarding the pups in the sham group, the right carotid artery was exposed without any further manipulations.

The pups were randomly allocated into five groups: sham, HIE+vehicle, HIE+Ang II, HIE+valsartan, and HIE+SB216763 groups (*n* = 6/group). The pups in the HIE+Ang II group were intracerebroventricularly administered Ang II (0.25 *μ*g/side) after cell modeling at 1 h and 24 h, pups in the HIE+valsartan group were gavaged valsartan (5 mg/kg/d) for ten days before HIE modeling, and pups in the HIE+SB216763 group were intracerebroventricularly administered SB216763 (20 *μ*g/kg) immediately after inducing HIE. The pups in the HIE+vehicle group were administered with the corresponding dissolvent with the same volume. The righting and geotaxis reflexes were measured, and body weight was also recorded at 48 h after HIE modeling. All the experimental procedures were approved by the Ethics Committee of Guizhou Provincial People's Hospital.

### 2.7. 2,3,5-Triphenyltetrazolium Chloride (TTC) Staining

TTC staining was performed as described in a previous study [36]. The pups were sacrificed via decapitation, and the brain was collected and frozen for 20 min at –20°C at 48 h after HIE. Next, a total of five 1.5 mm sequential coronary sections were cut and immersed in 1% TTC for 15 min at 37°C in the dark. Afterward, the sections were fixed using paraformaldehyde, which were then photographed and analyzed using Quantity One (Bio-Rad, USA). The infarction volume was calculated as the infarct area/total area of the cerebral section.

### 2.8. Statistical Analysis

Data were expressed as mean ± standard deviation (SD). The comparison among groups were analyzed using One-way analysis of variance (ANOVA) and least significant difference (LSD) post hoc analysis. *P* value less than 0.05 was considered statistically significant.

## 3. Results

### 3.1. Ang II Induced Neuronal Cell Death In Vitro

**Figure 2:**
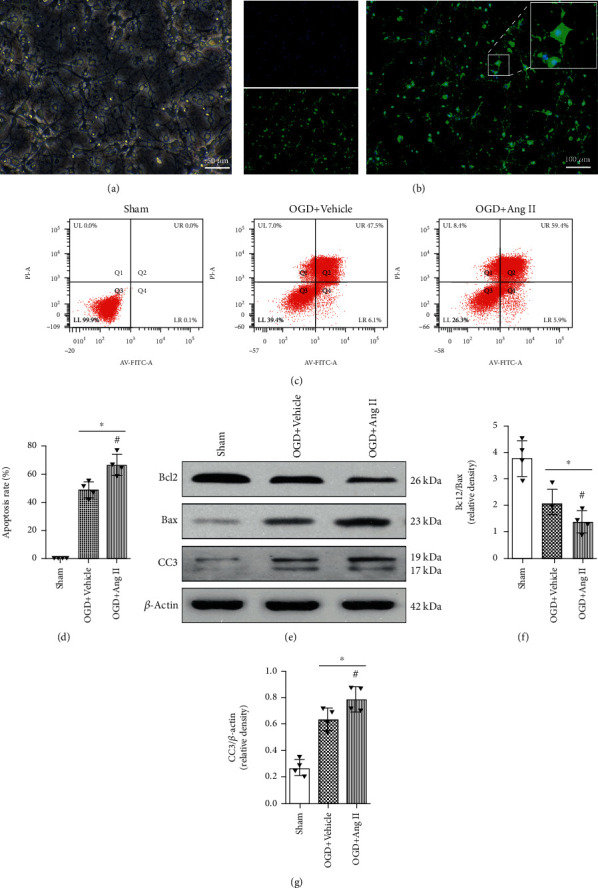
Ang II induced neuronal apoptosis. (a) The morphology of isolated primary neuronal cells, 100×. (b) IF for MAP2. Scale bar, 100 *μ*M. (c) Cell apoptosis detected by flow cytometry. (d) Calculation for the apoptotic cells. (e) Western blot. (f) Bcl-2/Bax ratio. (g) Band density for CC3 expression. CC3: cleaved caspase-3. ∗*P* < 0.05 vs. sham; ^#^*P* < 0.05 vs. OGD+vehicle.

The morphology of the isolated neuronal cells was detected via microscope, which presented with typical neuronal features (Figure 2(a)). Additionally, MAP-2 staining further proved that the isolated cells were neuronal cells (Figure 2(b)). Next, the cells were insulted using glucose-free Earle's Balanced Salt solution to establish the OGD cell model and were treated with Ang II. It was shown that when compared with OGD cells, Ang II significantly induced cell apoptosis (Figures 2(c) and 2(d)), and the expression of Bcl-2 was significantly downregulated, whereas the expression of Bax and cleaved caspase-3 was significantly increased (Figures 2(e) and 2(g)). The Bcl-2/Bax ratio was significantly downregulated after Ang II treatment (Figure 2(f)). These findings suggest that Ang II could induce neuronal cell death.

### 3.2. Ang II and AT1R Were Upregulated in HIE Rats

**Figure 3:**
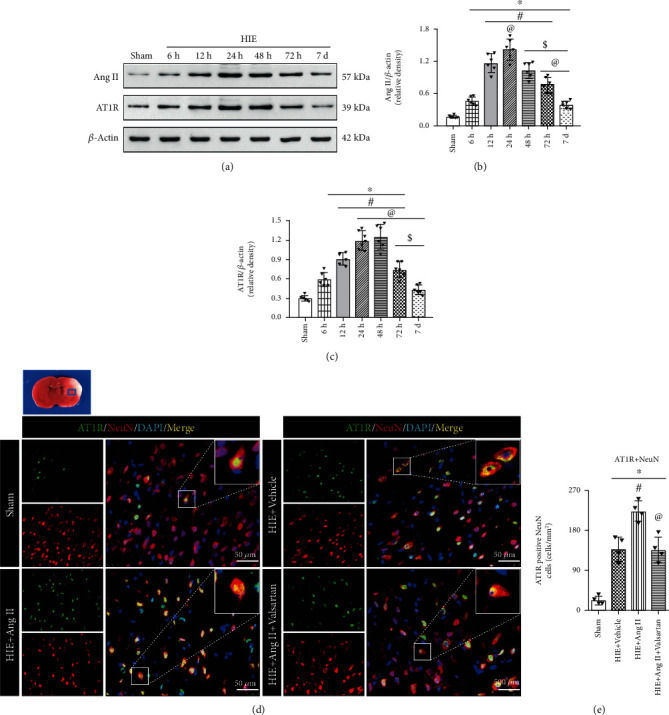
Ang II and AT1R were increased post-HIE establishment. (a) Western blot. (b, c) Band density for Ang II and AT1R. ∗*P* < 0.05 vs. sham; ^#^*P* < 0.05 vs. 6 h HI; ^@^*P* < 0.05 vs. 12 h HI; ^$^*P* < 0.05 vs. 24 h HI. *n* = 6 per group. (d) IF of AT1R on NeuN-positive cells. Scale bar, 100 *μ*M. (e) Quantitative analysis for AT1R and NeuN-double-positive cells. ∗*P* < 0.05 vs. sham; ^#^*P* < 0.05 vs. HIE+vehicle; ^@^*P* < 0.05 vs HIE+AngII. *n* = 6 per group.

Next, we sought to explore the expression level of Ang II and AT1R in vivo. As expected, the expressions of Ang II and AT1R were significantly increased and peaked at 24 h after HIE (Figures 3(a)–3(c)). Then, we determined whether Ang II could also induce neuronal cell death in vivo, and IF was used to detect AT1R expression in nearby ischemic brain tissues. As depicted in Figures 3(d) and 3(e), few NeuN-positive cells expressed AT1R in the sham group, and AT1R was significantly increased post-HIE. Ang II treatment further increased AT1R expression compared with the HIE modeling group, while Valsartan abrogated Ang II-induced AT1R augment (Figures 3(d) and 3(e)).

### 3.3. Valsartan or SB216763 Attenuated Brain Injury Induced by Ang II Post-HIE

**Figure 4:**
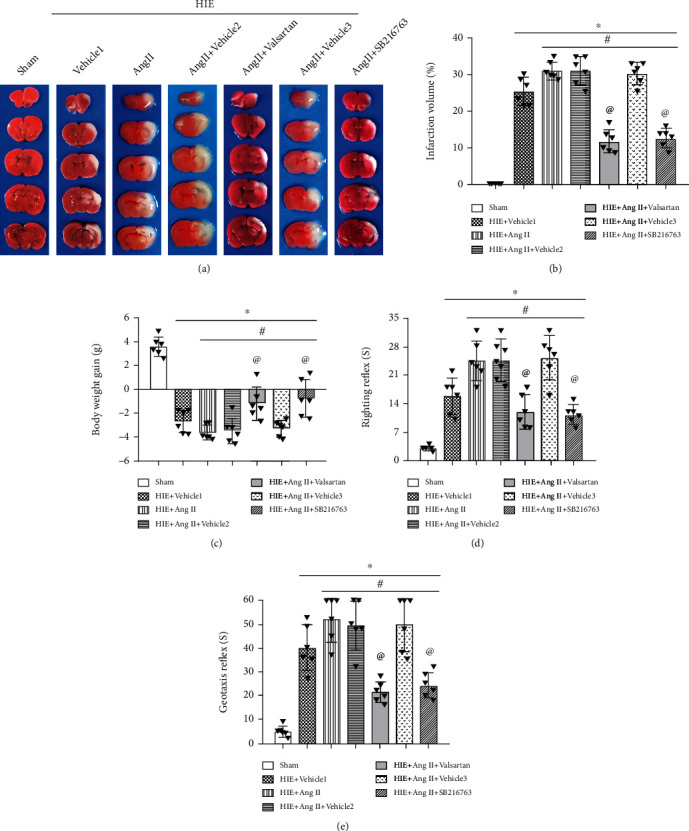
AT1R and GSK-3*β* inhibition reversed Ang II-induced infarct volume and neurological deficits at 48 h after HI. (a) TTC analysis. (b) Quantitative analysis for TTC staining of the cerebral infarction volume. (c) Body weight. (d) Righting reflex. (e) Geotaxis reflex. ∗*P* < 0.05 vs. sham; ^#^*P* < 0.05 vs. HIE+vehicle1; ^@^*P* < 0.05 vs. HIE+AngII. *n* = 6 per group.

**Figure 5:**
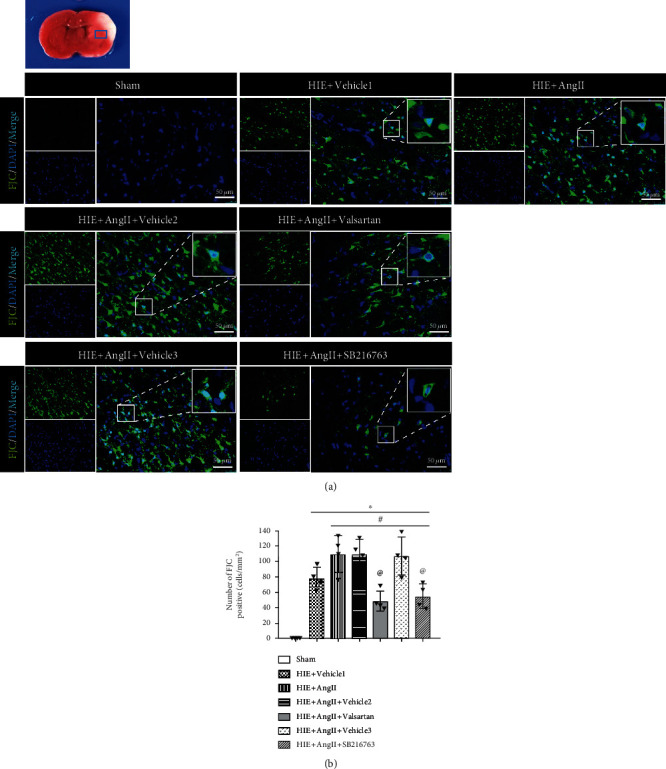
Neuronal degeneration. (a) FJC staining. (b) Quantitative analysis of FJC-positive cells. FJC: Fluoro-Jade C; ∗*P* < 0.05 vs. sham; ^#^*P* < 0.05 vs. HIE+vehicle1; ^@^*P* < 0.05 vs. HIE+AngII. *n* = 6 per group.

**Figure 6:**
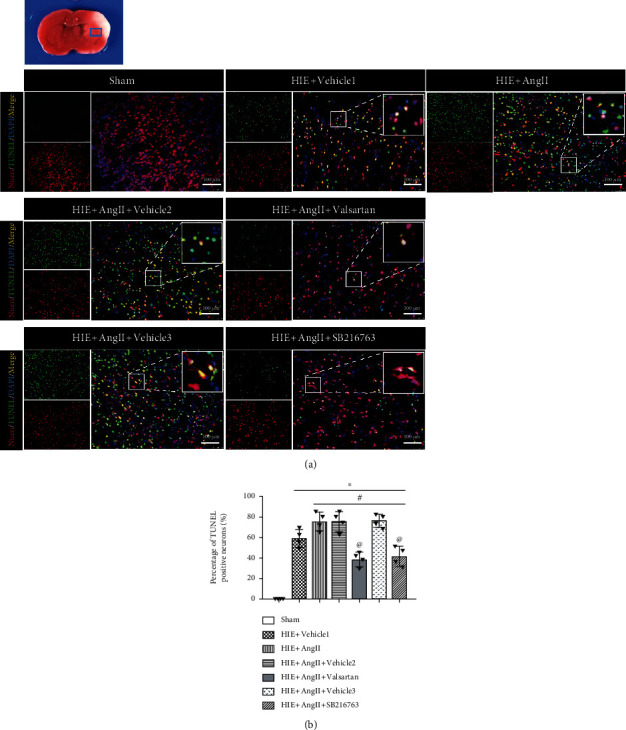
TUNEL assay for neuronal death. (a) TUNEL assay. (b) Quantitative analysis of apoptotic cells. TUNEL: terminal deoxynucleotidyl transferase-mediated dUTP-biotin nick end labeling. ∗*P* < 0.05 vs. sham; ^#^*P* < 0.05 vs. HIE+vehicle1; ^@^*P* < 0.05 vs HIE+AngII. *n* = 6 per group.

Afterward, we evaluated the effects of valsartan (AT1R inhibitor) and SB216763 (GSK-3*β*) on Ang II. It was shown that treatment with either valsartan or SB216763 led to a significantly reduced infarction volume compared with Ang II treatment alone (Figures 4(a) and 4(b)). Ang II increased the number of AT1R-positive neurons which was reversed by valsartan (Figures 3(d) and 3(e)). Moreover, we found that there was significant body weight loss after HIE, but valsartan and SB216763 each reversed body loss (Figure 4(c)). In addition, as shown in Figures 4(d) and 4(e), Ang II treatment significantly prolonged the righting reflex and geotaxis reflex, but valsartan and SB216763 each improved neurological deficits. Neuronal degeneration was considered an important indicator for brain-related diseases, hence, FJC staining was used to detect neuronal degeneration. As shown in Figure 5, the number of FJC-positive cells was significantly increased post-HIE, and Ang II further increased the number of degenerated cells. Consistent with TTC staining, valsartan or SB216763 significantly reduced the number of FJC-positive cells. Additionally, fluorescein-dUTP staining (TUNEL assay) showed similar results (Figure 6). These results suggest that valsartan or SB216763 reversed Ang II-induced neuronal damage in vivo.

### 3.4. AT1R Inhibition Reversed Ang II Induced Brain Damage via AT1R/GSK-3*β*/mTOR Axis

**Figure 7:**
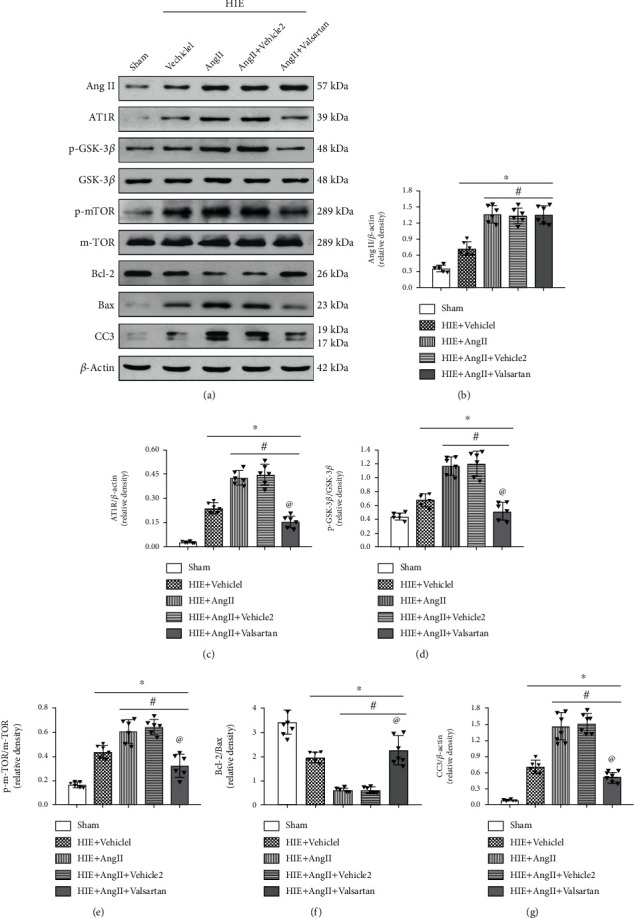
ATGR inhibition on Ang II induced cell death. (a) Western blot. (b) Band density for Ang II. (c) Band density for AT1R. (d) pGSK-3*β*/GSK-3*β* ratio. (e) p-mTOR/mTOR ratio. (f) Bacl-2/Bax ratio. (d) Band density for CC-3. CC-3: cleaved caspase-3; ∗*P* < 0.05 vs. sham; ^#^*P* < 0.05 vs. HIE+vehicle1; ^@^*P* < 0.05 vs HIE+AngII. *n* = 6 per group.

Next, we sought to detect the underlying mechanism by which Ang II-mediated brain damage. As shown in Figures 7(a)–7(c), Ang II-induced AT1R expression and valsartan abrogated the induction. The expressions of mTOR and GSK-3*β* remain unchanged. However, the phosphorylation of mTOR and GSK-3*β* was significantly upregulated by Ang II, and valsartan significantly reduced their expression (Figures 7(a), 7(b), 7(d), and 7(e)). Moreover, compared with Ang II, valsartan also increased the Bcl-2/Bax ratio, while decreasing cleaved caspase-3 expression (Figures 7(a), 7(b), 7(f), and 7(g)). These results suggest that AT1R blockade reversed Ang II-induced brain injury, in which GSK-3*β*/mTOR was involved.

**Figure 8:**
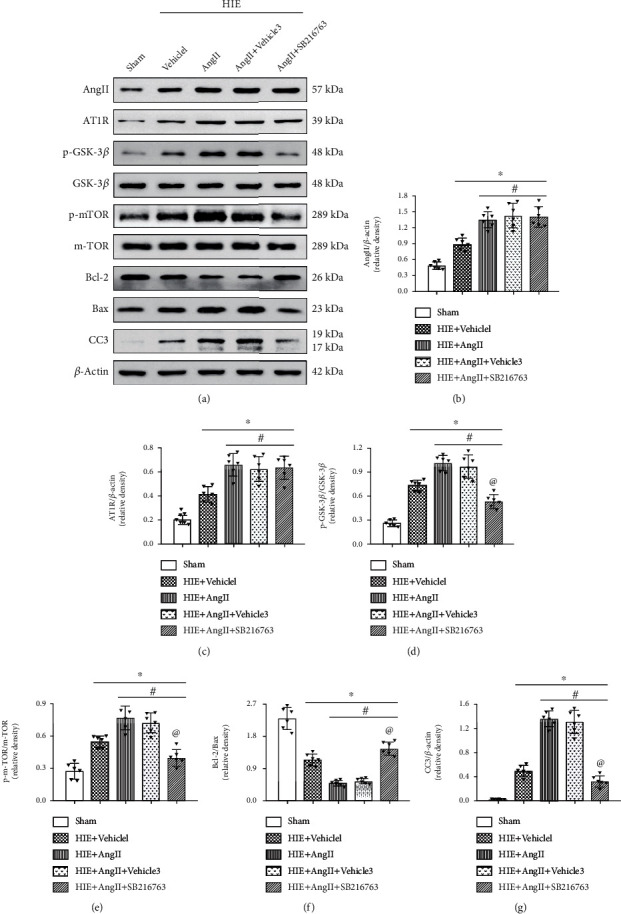
GSK-3*β* inhibition on Ang II induced cell death. (a) Western blot. (b) Band density for Ang II. (c) Band density for AT1R. (d) pGSK-3*β*/GSK-3*β* ratio. (e) p-mTOR/mTOR ratio. (f) Bacl-2/Bax ratio. (g) Band density for CC-3. CC-3: cleaved caspase-3; ∗*P* < 0.05 vs. sham; ^#^*P* < 0.05 vs HIE+vehicle1; ^@^*P* < 0.05 vs. HIE+AngII. *n* = 6 per group.

To further evaluate the role of GSK-3*β*/mTOR in Ang II-induced brain injury, SB216763 was also utilized. It was shown that SB216763 significantly suppressed the expression of phosphorylated mTOR and GSK-3*β* at 48 h after HI in the HIE+SB216763 group compared with the HIE+SB216763+vehicle group; SB216763 did not change the expression of AT1R compared with Ang II (Figures 8(a)–8(e)). Furthermore, compared with Ang II, SB216763 also increased the Bcl-2/Bax ratio and decreased cleaved caspase-3 expression (Figures 8(a), 8(b), 8(f), and 8(g)). These findings strongly indicated that AT1R/GSK-3*β*/mTOR were possibly the primary downstream mediators for Ang II-induced brain damage.

## 4. Discussion

In the present study, we found that Ang II was increased in HIE rats, and Ang II induced neuronal cell death both in vitro and in vivo. The blockade of AT1R or GSK-3*β* could ameliorate the adverse effects caused by Ang II, indicating that AT1R/GSK-3*β*/mTOR mediated Ang II-induced brain damage. These findings revealed the role and mechanism of Ang II in neuronal pathology and provided a potential drug target for HIE treatment.

The role of Ang II-induced hypertension has been extensively evaluated [37]. However, in recent years, Ang II's role beyond hypertension has been extensively analyzed, such as its role in neuroscience. For example, Wu et al. found that Ang II could induce cardiac remodeling [38]; Fan et al. revealed that Ang II promoted capillary damage in cerebral vasculature [39]. Additionally, Liu et al. reported that Ang II was an important mediator in the pathogenesis of diabetes, obesity, and hyperlipidemia and also regulates apoptosis, proliferation, autophagy, and insulin resistance [40]. Moreover, several studies also revealed that Ang II could increase cerebral hypoperfusion-induced anxiety-like behavior and promote memory impairment [41], induce neuronal dysfunction [42], contribute to the pathogenesis of Alzheimer's Disease [43], cause neuronal damage [44] hippocampal neural stem cell death, and memory impairment [45]. In addition, an increasing number of studies have found that RAS plays an important role in the occurrence and rehabilitation of stroke cerebrovascular diseases [46–48]. After binding to its receptors, Ang II can activate a series of cell signaling pathways, including phosphatidyl inositol (PI) signaling pathways associated with the vasoconstrictor function of Ang II. For example, second messengers have been shown to facilitate the binding of Ang II to AT1R to promote downstream effects. When Ang II acts on AT1R, it can cause local vasoconstriction, blood flow interruption, inflammatory reactions, and reactive oxygen species production, which could lead to neuronal apoptosis. In addition to the interaction with G proteins, AT1R also activates intracellular pathways, such as MAPK and JAK/STAT mechanisms. Ang II binding to AT1R also caused phosphorylation of PLC*γ*, which cleaves PIP2 to produce IP3 and DAG, followed by Ca^2+^ mobilization and protein kinase C (PKC) activation. These second messengers generated through AT1R then contributed to the vasoconstrictor function of Ang II, as well as activation of downstream tyrosine and serine/threonine kinases, which contributes to the growth-promoting and cytokine-like actions of Ang II [49, 50]. Long-term blockade of AT1R reportedly improves neurological outcome and reduces the infarct volume after experimental focal cerebral ischemia [51]. In agreement with these findings, the present study found that Ang II induced neuronal cell death, promoted neuronal degeneration, and enlarged the infarction volume in HIE rats and the OGD cell model. In conjunction with other studies, we concluded that Ang II might play an important role in the pathogenesis of neuronal diseases.

GSK-3*β* has been shown to play vital roles in various biological processes and has shown to be a downstream mediator for Ang II. Narasimhan et al. reported that inhibiting OBG-like ATPase alleviated the Ang II/GSK-3*β*-induced hypertrophic response [30, 52]. In addition, Ang II-mediated GSK-3*β* signaling promoted muscle waste [53]. In the present study, we discovered that Ang II/AT1R could also regulate GSK-3*β* in mediating neuronal degeneration and death, which broadened our understanding that Ang II could activate GSK-3*β* in brains. The activation of GSK-3 has been proven to phosphorylate its downstream components and regulate their activity [54]. It is widely accepted that mTOR was the downstream mediator for PI3k/Akt signaling, and GSK-3 orchestrated PI3k/Akt signaling to regulate biological functions. Therefore, in this study, we evaluated whether GSK-3 inhibition could alter mTOR activity. As expected, we found that the application of SB216763 abrogated phosphorylation of GSK-3*β* and mTOR and ameliorated brain damage, indicating that the GSK-3*β*/mTOR cascade mediated Ang II-triggered brain injury. Furthermore, the inhibition of AT1R also blocks GSK-3*β* and mTOR phosphorylation and the phenomena caused by Ang II. These results strongly suggested that the AT1R/GSK-3*β*/mTOR cascade participated in Ang II-induced neuronal characteristic alteration. More importantly, we should note that valsartan, commonly used in clinical application, warrants further clinical investigation for its potential in HIE treatment.

## 5. Conclusions

Taken together, the present study revealed that Ang II mediated AT1R/GSK-3*β*/mTOR signaling, having a role in HIE pathogenesis and the blockade of AT1R or GSK-3*β*, could protect neurons from apoptosis and degeneration. Future analyses are necessary to unveil whether PI3K/Akt was involved in this process.

## Data Availability

The data support the findings of this study and are available from the corresponding author upon reasonable request.
